# What has preclinical systematic review ever done for us?

**DOI:** 10.1136/bmjos-2021-100219

**Published:** 2022-03-12

**Authors:** Ash Allanna Mark Russell, Brad A Sutherland, Lila M Landowski, Malcolm Macleod, David W Howells

**Affiliations:** 1 Tasmanian School of Medicine, College of Health and Medicine, University of Tasmania, Hobart, Tasmania, Australia; 2 School of Health Sciences, College of Health and Medicine, University of Tasmania, Hobart, Tasmania, Australia; 3 Centre for Clinical Brain Sciences, Edinburgh Medical School, The University of Edinburgh, Edinburgh, UK

**Keywords:** systematic review, meta-analysis, preclinical

## Abstract

Systematic review and meta-analysis are a gift to the modern researcher, delivering a crystallised understanding of the existing research data in any given space. This can include whether candidate drugs are likely to work or not and which are better than others, whether our models of disease have predictive value and how this might be improved and also how these all interact with disease pathophysiology.

Grappling with the literature needed for such analyses is becoming increasingly difficult as the number of publications grows. However, narrowing the focus of a review to reduce workload runs the risk of diminishing the generalisability of conclusions drawn from such increasingly specific analyses.

Moreover, at the same time as we gain greater insight into our topic, we also discover more about the flaws that undermine much scientific research. Systematic review and meta-analysis have also shown that the quality of much preclinical research is inadequate. Systematic review has helped reveal the extent of selection bias, performance bias, detection bias, attrition bias and low statistical power, raising questions about the validity of many preclinical research studies. This is perhaps the greatest virtue of systematic review and meta-analysis, the knowledge generated ultimately helps shed light on the limitations of existing research practice, and in doing so, helps bring reform and rigour to research across the sciences.

In this commentary, we explore the lessons that we have identified through the lens of preclinical systematic review and meta-analysis.

## Introduction

**Figure 1:**
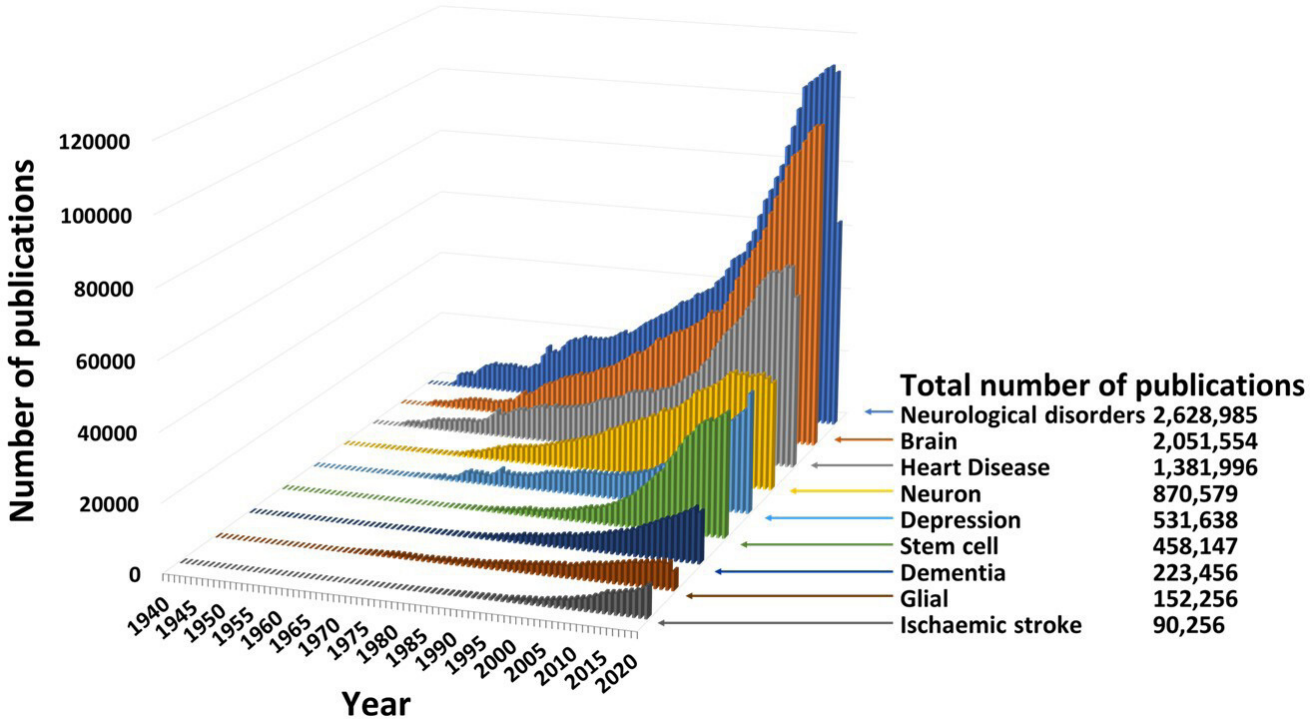
Results of PubMed search performed on 3 March 2021 showing numbers of publications by year and totals for subject searches using ‘MeSH’ headings.

The rate of growth of knowledge claims and the literature in which these are communicated is now so great that we are faced with major data overload. For example, in the field of neurological disorders, more than 2.6 million papers were classified as relevant by PubMed ‘MeSH’ headings. Thousands more publications describe other similar fields of research (figure 1). No individual can read, let alone absorb and master all this detail, so we rely on summaries to gain an overview of a topic.

The traditional approach to summarising the literature has been the narrative review, where (at least conceptually) an expert, or group of experts, condenses the knowledge gained over one, or many lifetimes of work in a field. Journals value these reviews because they highlight areas of current development and attract many citations, raising the profile of the journal. Junior researchers like them because they provide a short-cut into a complex world they are just beginning to explore. Despite their undoubted value as important repositories of human wisdom, narrative reviews have limitations. In most instances, the literature described represents only a small proportion of the total evidence available, and it is rare for the reader to be able to discern the reasons for the selection of some papers and exclusion of others. It is also rare for narrative reviews to provide a quantitative summary of the underlying data to justify the conclusions which they reach. In both instances, we are forced to trust the judgement of the narrative review authors but are unable to judge the scientific basis of the claims made. An unacknowledged problem is that writing such reviews is a task often given to PhD students and junior post-doctoral researchers on starting a new project. While a great training tool, they cannot truly be considered expert reviews.

Systematic review and meta-analysis try to address these limitations. By clearly defining the scope of the review and providing replicable search criteria, their readers know explicitly what subject matter was assessed. By providing inclusion and exclusion criteria, systematic reviews also tell the reader what was considered important and what was not. Moreover, where performed, meta-analyses provide a formal environment for testing the statistical validity of claims made by the included literature. This includes assessment of whether or not publication bias might contribute to a falsely positive picture of a subject. By embracing the principles of Open Science,^1^ systematic reviews and meta-analyses have the additional advantage of permitting ongoing aggregation of data, an important consideration when faced with an ever-growing literature. However, this comes at a cost to the researcher. Because the process requires current searching and assessment of what is often a large body of literature, it is a demanding and time-consuming task. Consequently, systematic reviews usually have a very narrow focus and lack breadth. Ultimately, we need meta meta-research (meta-analysis of meta-analyses) to integrate these high-quality yet narrow snippets of information into research outputs with broader applicability. It should be remembered, however, that systematic review and meta-analysis contain a risk of error caused by the rigid application of procedure and that not all knowledge is a matter of data aggregation.

Importantly, asking pertinent questions, establishing effective criteria for inclusion and exclusion of publications and deciding what data to extract from a body of literature still requires expertise. It should also be noted that systematic reviews are not immune from human foibles. The principle of ‘garbage in, garbage out’ still applies. If this is recognised, systematic review and meta-analysis can provide a vehicle for assessing the consequences of poor science and may salvage important signals that might otherwise be missed if suboptimal science is discarded outright.

In this commentary, we discuss some of our key observations from the formative years of preclinical systematic review and meta-analysis research. We present these in the form of ‘lessons’ (box 1). These lessons are not about the scientific aims or conclusions drawn by individual papers but about the environment in which they are performed. We acknowledge that these might one day become the specific subjects of new systematic reviews and meta-analyses but to do so is beyond the scope of this commentary. Nevertheless, in the spirit of systematic review, where we provide examples to illustrate these lessons, we have selected data in an unbiased and transparent way.

Box 1 **Summary of the lessons that have emerged from preclinical systematic review and meta-analysis**

1. Systematic review can compare interventions and assess preclinical models.
2. Literature overload limits research productivity.
3. Electronic databases have very different coverage.
4. Not all publications have value for meta-research.
5. The internal validity of many preclinical experiments is poor.
6. Publication bias is common.
7. Small preclinical studies present problems.
8. Promising reviews are rarely followed-up by their authors.

### Lesson 1: systematic review can compare interventions and assess preclinical models

In most cases, systematic review and meta-analysis in the preclinical space are performed to ask the question ‘are we there yet?’. Is the preclinical data sufficient to support a move into the clinical sphere? A recent cross-sectional study (2015–2018) examining the epidemiology and reporting characteristics of preclinical systematic reviews found that 54% assessed pharmacological interventions and 46% assessed non-pharmacological (mainly cellular or surgical) interventions across 23 different disease domains.^2^ However, many studies do not just ask whether a single intervention works. Many also examine the relative efficacy within classes of interventions, how varying conditions employed during modelling modify the apparent efficacy of an intervention, and the validity of preclinical models themselves.

For example, a broad systematic review examining the efficacy of 1026 experimental treatments for stroke found that the best candidates were not always those taken to clinical trial. Additionally, as the breadth of experimental testing increased, effectiveness appeared to decrease.^3^ Another stroke study found that while systematic review supported treatment of ischaemic stroke with magnesium (anti-glutamatergic), melatonin (anti-oxidant) and minocyclin (anti-inflammatory), appropriately powered, randomised and blinded experiments across a range of ischaemic conditions were unable to detect any efficacy.^4^ This raises concerns about the true efficacy of many of the candidates that appear promising in systematic reviews. Conversely, in models of multiple sclerosis, systematic review has identified drugs effective across multiple outcome domains which have not been tested in humans suggesting clinical evaluation of these otherwise neglected drugs may be of value.^5^

Systematic review and meta-analysis have also been used to explore the experimental conditions most conducive to detection of benefit. For example, for stem cell therapies in renal disease, cardiac disease, stroke and spinal cord injury, therapeutic efficacy is inversely related to the size of the experimental animal, raising important questions about the doses that might be required for effective translation to humans.^6^ Others have used systematic review to support the face validity of models of, for example, cerebral palsy^7^ and xenograft models of colorectal cancer,^8^ or to justify calls for greater standardisation in preclinical experiments.^9^

### Lesson 2: literature overload limits research productivity

The inclusivity of systematic reviewing presents a specific challenge, the person-power needed to find and sift publications for inclusion within the review. A utopian view would require us to perform broad reviews that examined the entire data set of a field of research. This would enable comparison of issues such as the strengths and weaknesses of our model systems, the relative merits of candidate drugs used within such models or the impact of interacting variables often present but not controlled for, such as anaesthesia in surgical models or staffing interactions during behavioural testing. Such broad studies are rare and probably unrealistic for most research teams until all publications are available in a searchable electronic format and automated text and data extraction tools mature. Asking a broad research question finds more publications than are easy to handle. As in science in general, asking a more specific question reduces the potential workload but at the probable expense of generalisability.

**Figure 2:**
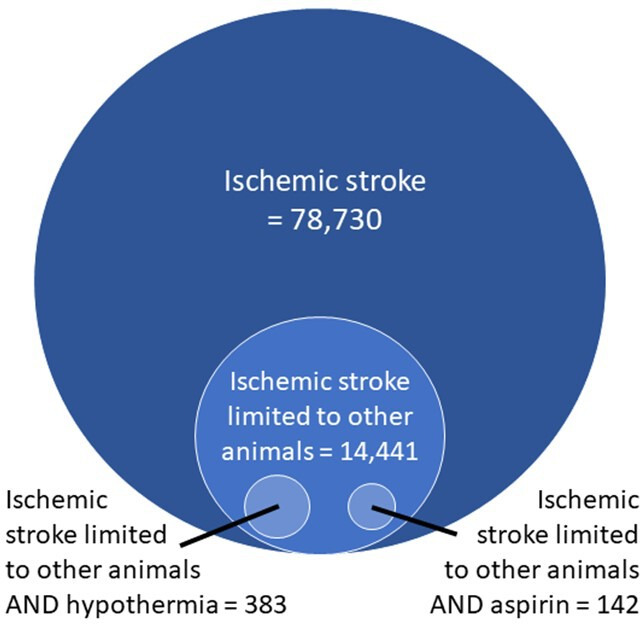
Results of simple searches performed in PubMed illustrating potential workload for systematic review and meta-analysis. Search completed on 19 January 2021 using the ‘advanced’ search option on the front page and the ‘species’ filter on the search results page.

To illustrate this, we completed a simple and easily replicable search using PubMed’s ‘advanced’ search filter (figure 2). By searching for the neurological disorder ‘ischaemic stroke’ we can see that a large number of potentially relevant papers are found, much larger than any one reviewer could handle alone (78 730 total). Limiting the search to studies containing animals only and to studies listing particular treatments (hypothermia or aspirin), we reduce the number of studies identified with our search to a few hundred. This number is much more tractable for a systematic review project, but there is a risk that relevant papers are being missed in these narrower searches. That is, the increased specificity comes at the costs of some reduction in sensitivity.

We do not argue for less primary research but do argue that making such data more accessible for meta-research will improve future productivity and understanding. Importantly, consideration of logistics is critical when planning a systematic review. Automation tools are increasingly useful adjuncts to human effort, but the human effort is still substantial.

### Lesson 3: electronic databases have very different coverage

Finding the publications would seem to be a simple task; after all, many electronic repositories/directories of the scientific literature are available for this purpose. However, no search engine provides access to a complete record of all published works. Search results with different tools vary with respect to subject matter and period of coverage, or how the indexing works (use of proprietary search and indexing algorithms and human curation).^10 11^ Changes to any of these databases or their indexing systems without public documentation also means that full reproducibility during future studies cannot be assured. Systematic reviewers must balance scientific purity against pragmatism.

Depending on your branch of science, you may have access to many literature databases. In our field, PubMed, EMBASE, Scopus (the latter two both owned by Elsevier) and Web of Science/BIOSIS are commonly used. While all cover biomedical and life science literature, their coverage differs substantially.^11^

To illustrate these issues of coverage and address additional questions below, we performed a simple search to find systematic reviews of preclinical therapeutic interventions to determine where these reviews found their primary data. On 3 March 2021, PubMed indexed 180 585 publications as systematic reviews. Of those with ‘Free full text’ available, PubMed’s inbuilt ‘other animal’ filter identified 5593 (3%) as potentially relevant to animal research. One thousand five hundred and seventy-two (0.9% of all PubMed returns, 28% of those filtered as ‘other animal’) self-identified as a systematic review, including the term in the article title. On inspection of these papers, we found 283 papers examining therapeutic interventions in preclinical disease models. We selected 20 of these at random for closer examination,^12–31^ their characteristics are summarised in table 1.

**Table 1:** Characteristics of 20 randomly selected systematic reviews, including the disease or biological system of interest and the treatment/intervention being investigated. Total number of studies identified through systematic searches and number of studies included in each review’s final analysis were self-reported in each publication, percentage of studies included out of all studies identified was calculated by the authors of this paper

Study (reference)	Disease/biological system	Treatment/intervention	# studies identified from searches	# studies included in final analysis	% studies included in final analysis
Albuquerque *et al*^12^	Melanoma	Plant extracts	1359	35	2.6
Archambault *et al*^13^	Neonatal hypoxic ischaemic encephalopathy (HIE)	Mesenchymal stem/stromal cells (MSCs)	161	18	11.2
Ashcraft *et al*^14^	Cancer	Aerobic exercise	466	53	11.4
Auboire *et al*^15^	Ischaemic stroke	Microbubbles (MBs) combined with ultrasound sonothrombolysis (STL)	2506	16	0.6
Cao *et al*^16^	Gut microbiota	Anti-hyperglycaemic drugs	4075	64	1.6
Chen *et al*^17^	Ischaemic stroke	Neural stem cells (NSCs) transplantation therapy	2524	37	1.5
Dong *et al*^18^	Ischaemic stroke	Recombinant tissue plasminogen activator (rtPA)	2128	47	2.2
Gaubys *et al*^19^	Regeneration of periodontal tissue complex	Stem cell therapy	2099	10	0.5
Janssen^20^	Ischaemic stroke	Constraint-induced movement therapy (CIMT)	3580	8	0.2
Lambrecht^21^	Anaemia	Animal husbandry and capture (AHC)	9027	23	0.3
Li *et al*^22^	Hepatocellular carcinoma	Metformin	573	13	2.3
Liao *et al*^23^	Injury to bone	Stem cell therapy	202	20	9.9
Ma *et al*^24^	Ischaemic stroke	Xingnaojing injection (XNJI)	392	23	5.9
Senders *et al*^25^	Glioma surgery	Agents for fluorescence-guided glioma surgery	2619	105	4.0
Silverblatt *et al*^26^	Myocardial injury	Beta blockers, calcium channel blockers and antagonists of the renin–angiotensin system	347	52	15.0
Suen *et al*^27^	Pulmonary arterial hypertension (PAH)	Regenerative cell therapies	1368	45	3.3
van der Bent *et al*^28^	Heritable neurodegenerative and neuromuscular diseases	Antisense oligonucleotide (AON)-based therapies	1330	95	7.1
van der Spoel *et al*^29^	Ischaemic heart disease	Stem cell therapy	304	52	17.1
Wei *et al*^30^	Ischaemic stroke	Buyang Huanwu decoction (BHD)	973	56	5.8
Zhang *et al*^31^	Vascular dementia	Acupuncture	194	16	8.2

**Figure 3:**
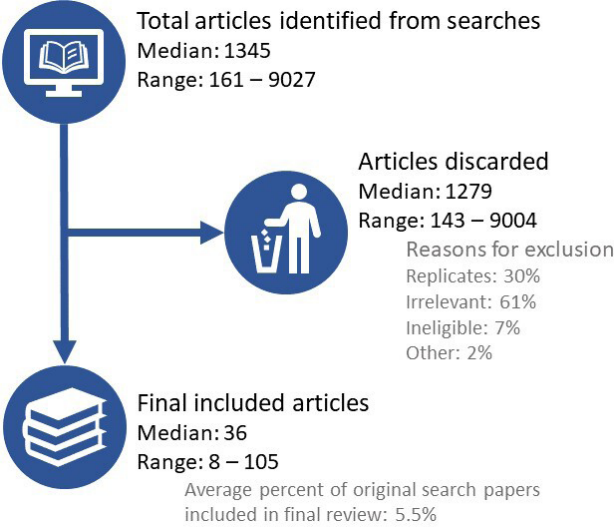
Screening data (extracted from PRISMA diagrams or text) from 20 randomly selected systematic reviews published between 2011 and 2020. Of the original 20 papers, three were excluded and replaced as the PRISMA diagrams contained mathematical errors. ‘Reasons for exclusion’ are expressed as a mean percentage of each review’s excluded publications. ‘Replicates’=studies found in multiple databases, only a single replicate is included in further screening and all other replicates are discarded, ‘irrelevant’=did not answer the research question asked by this review, ‘ineligible’=did not contain data useable by this review, ‘other’=papers excluded for reasons such as not having an available full text, being written in languages not included in the review or being a publication other than a primary research article. PRISMA, Preferred Reporting Items for Systematic Reviews and Meta-Analyses.

In these 20 studies, the authors had searched using the name of the intervention of interest within a broader pool of papers specific to a disease model in non-human animal species, giving little scope for ambiguity. The median number of databases searched by these reviews was 3 (range 1–7). The most commonly searched databases were PubMed, EMBASE and Web of Science. Examining the reason why publications were excluded from each review’s analysis shows that on average, 30% (range 0%–92.3%) of a review’s discarded papers were replicates found across multiple databases (median 214 replicates per review) (figure 3). The corollary of this is that many unique and potentially important papers would be missed if only a single database had been searched. The magnitude of this problem, and the extent to which adding a further database to be searched changes the conclusions of a meta-analysis, is unclear. Luijk^32^ found that for some reviews the global estimate of efficacy was lower when limited to studies identified through PubMed compared with all other databases; from PubMed and EMBASE compared with all other databases; and when inclusion was limited to English language publications. However, overall, there was no significant effect. Information scientists have been aware of such problems for decades. We should also remember—in the context of systematic reviews that aspire to assess all available data on a subject—that the English literature is not the only source of scientific data and it is critical that such language bias is avoided.^33^

### Lesson 4: not all publications have value for meta-research

Among several thousand publications found by electronic searching, examination of the search results and application of the systematic review inclusion and exclusion criteria also illustrates that the returns of electronic searches do not always provide a high yield of relevant data. We examined the total identified, excluded and included studies in our 20 randomly selected reviews (table 1) and noted the reasons nominated by review authors for their exclusions (figure 3). In the 20 reviews, each identified a large number of papers that might have been relevant (median 1345 publications identified per review, range 161–9027). However, on average, only 5.5% (range 0.2%–17.1%, median 36 publications per review) of these potentially relevant papers were included in each review’s final data set and their analyses. If we consider the other 94.5% of identified publications which were excluded per review (range 82.9%–99.8%, median 1279 excluded publications), we find that 61% of them (range 7.1%–99.9%, median 624 publications per review) were excluded as they were considered irrelevant when the abstracts were read by a screening team. Additionally, an average of 7% (range 0%–15.5%, median 15.5 publications) of each review’s excluded publications were excluded by the review authors because of omissions in data reporting. Usually this takes the form of reporting outcome measures that are not usable for meta-analysis or missing important parameters in the describing of methods. From the perspective of a systematic reviewer, this is frustrating. An inordinate amount of effort goes into evaluation of papers of little value. One could argue this is highly dependent on the sensitivity/specificity of the search terms used. However, it should be remembered that we selected the data for figure 3 because the authors of these systematic reviews were asking relatively unambiguous questions about very specific interventions. It is likely that studies asking more complex and ambiguous questions might have larger initial searches and exclude even more studies. This strongly suggests that better sensitivity and specificity require improvements to the indexing provided by the respective databases and the clarity of titles, abstracts and key words provided by originating authors.

The inclusion of articles that are subsequently discovered to be fraudulent may also contribute to a systematic review if the review is performed prior to the fraudulent discovery. However, it is outside the scope of the reviewer to detect this given the lack of access to raw data, except for circumstances where individual subject data is used. The time of the systematic reviewer is of the least concern here, when set against the grant funding gone to waste, the exposure of animals to harms with little benefit in knowledge in return, and the efforts of research teams squandered by poor reporting.

### Lesson 5: the internal validity of many preclinical experiments is poor

As stated by the late Doug Altman and colleagues ‘If the raw material is flawed then the conclusions of systematic reviews cannot be trusted’.^34^ The internal validity of a study is threatened by bias, ‘any process at any stage of inference tending to produce results that differ systematically from the true values’.^35^ In preclinical studies, as in clinical trials, we see selection bias, performance bias, detection bias and attrition bias. Moreover, most publications fail to include information that would allow a reader to know to what extent biases might have been prevented.^4 36 37^ Within the preclinical literature, a variety of tools have been used to assess overall quality and risk of bias. Within the 20 preclinical systematic reviews we randomly selected as illustrative of trends within the field, the majority used either the Collaborative Approach to Meta Analysis and Review of Animal Data from Experimental Studies (CAMARADES) quality score checklist^38^ or SYstematic Review Center for Laboratory animal Experimentation’s (SYRCLE) risk of bias tool.^39^ Two studies made no assessment of study quality or risk of bias. Worryingly, only one study assessed both, which raises the possibility that the other 17 studies were unaware that assessing a study’s quality and assessing the study’s risk of bias are not the same thing (table 2). Risk of bias tools assess a publication for the potential presence of specific biases (listed above). Quality scores assign yes/no answers to questions regarding disease model specific norms, randomisation, blinding, reporting ethics approvals or conflicts of interest to assess a paper’s overall adherence to good research practices. For each of the systematic reviews we assessed, included publications scored poorly against either of these types of tool. The highest median score using SYRCLE’s risk of bias tool was 5 out of 10, which is much lower than desirable. Reviews reporting a quality score were similar. No review using the CAMARADES quality rating scored a single included publication higher than 8 out of 10, with the median score being 4.

**Table 2:** Median and range for quality/RoB scores were calculated for the studies included in each review

Study (reference)	Assessed quality/RoB	Score for included publications, median (range)	Scoring system
Auboire *et al*^15^	Yes—Quality	4.5 (3–6)	CAMARADES
Chen *et al*^17^	Yes—Quality	5 (3–7)	CAMARADES
Dong *et al*^18^	Yes—Quality	4 (2–6)	CAMARADES
Janssen *et al*^20^	Yes—Quality	5 (2–6)	CAMARADES
Liao *et al*^23^	Yes—Quality	2.5 (1–4)	Jadad scale (modified)
Ma *et al*^24^	Yes—Quality	4 (3–6)	CAMARADES
van der Spoel *et al*^29^	Yes—Quality	1 (0–5)	Authors' custom scale
Wei *et al*^30^	Yes—Quality	3 (2–6)	CAMARADES
Zhang *et al*^31^	Yes—Quality	5.5 (4–8)	CAMARADES
Silverblatt *et al*^26^	Yes—Both	5 (3–8)	CAMARADES (modified)
		5 (1–10)	SYRCLE’s Risk of Bias Tool
Albuquerque *et al*^12^	Yes—RoB	62.9 (40–74.3)	ARRIVE
Archambault *et al*^13^	Yes—RoB	4 (4–8)	SYRCLE’s Risk of Bias Tool
Cao *et al*^16^	Yes—RoB	3 (2–4)	SYRCLE’s Risk of Bias Tool
Gaubys *et al*^19^	Yes—RoB	3 (1–4)	Cochrane’s Risk of Bias Tool
Lambrecht *et al*^21^	Yes—RoB	2 (1–4)	GRADE (modified)
Li *et al*^22^	Yes—RoB	1.5 (1–5)	SYRCLE’s Risk of Bias Tool
Suen *et al*^27^	Yes—RoB	1 (0–2)	SYRCLE’s Risk of Bias Tool
van der Bent *et al*^28^	Yes—RoB	2 (0–8)	SYRCLE’s Risk of Bias Tool
Ashcraft *et al*^14^	No	–	–
Senders *et al*^25^	No	–	–

CAMARADES score is out of 10, Jadad scale is out of 5, SYRCLE’s Risk of Bias Tool is out of 10, ARRIVE score is a percentage, Cochrane’s Risk of Bias Tool is out of 7, GRADE score is out of 5, van der Spoel’s (2011) custom scale is out of 5.

ARRIVE, Animal Research: Reporting of In Vivo Experiments; CAMARADES, Collaborative Approach to Meta Analysis and Review of Animal Data from Experimental Studies; GRADE, Grading of Recommendations Assessment, Development, and Evaluation; RoB, Risk of bias; SYRCLE, SYstematic Review Center for Laboratory animal Experimentation.

While it can be argued that these poor assessments merely reflect a failure to report these issues and is of little consequence, the data suggest otherwise. Where systematic reviews have been able to stratify data by the presence or absence of randomisation and blinding (which should guard against bias), the detected effect sizes are often substantially reduced by the presence of these measures.^38 40–46^ Further, the increase in reporting which followed implementation of a change in editorial policy at nature was largely due to an increase in researchers reporting that they had not randomised, had not blinded or had not conducted a sample size calculation.^47^

### Lesson 6: publication bias is common

Publication bias (also known as dissemination bias) is the phenomenon whereby studies with positive results are more likely to be published than research with negative or neutral results, causing an over-representation of positive findings in the literature. This distorts that literature, making interpretation and assignment of value to knowledge claims difficult. In 2011, a systematic review of over 4600 research papers from different disciplines found that the frequency of publishing positive findings rose by over 22% between 1990 and 2007, with the fields of clinical medicine, pharmacology and toxicology, and molecular biology the worst affected.^48^

In preclinical stroke research, estimates of the proportion of missing neutral or negative studies ranged from 5% to 36% for different candidate therapies and across the field this constituted an overstatement of efficacy (relative) of 31%.^49^ Excess significance (where there are more statistically significant results than should be expected) has been detected in most preclinical systematic reviews in neuroscience^50^ and publication bias probably exists in most of the preclinical literature.^51^ A 2019 Nature commentary co-signed by over 800 statisticians discussed that of these statistically significant papers dominating the literature, 51% were likely wrongly interpreted, and most of them likely interpreted their results with the false belief that a significant p value determines whether a result is ‘real’ or not.^52^ Because systematic reviews should find all the available data, a biased literature will lead to a biased synthesis of that evidence unless statistical methods are used to identify and where possible correct for this problem.^50 53^ However, despite systematic reviews being well positioned to take advantage of their comprehensive data collection to check for bias, Mueller and colleagues found that only 50% of preclinical systematic reviews chose to assess publication bias in their analysis.^54^ Of the 20 studies we analysed for this commentary, only 11 (55%) considered publication bias.

The presence of publication bias is important. New studies using biased foundational data run the risk of unnecessary repetition of futile science that has been performed but not reported. This wastes valuable resources, leads to unethical use of animals and ultimately puts humans at risk if, consequently, they are recruited into misguided clinical trials.^55 56^

Among the standard tools in assessment of publication bias are the funnel plot, and derivatives such as Egger’s regression. These detect asymmetry in the distribution of the effect size of a result compared with a measure of its precision. In the absence of bias, the distribution is ‘funnel’ shaped and symmetrical about its mean with small imprecise studies, most influenced by random variation, distributed broadly towards the base. When publication bias is present, smaller, less precise studies, reporting negative or neutral results, will be missing and the distribution becomes skewed.^57^

### Lesson 7: small preclinical studies present problems

In 3145 preclinical stroke studies undertaken on 45 476 animals, the average cohort size was 7 animals per treatment group.^58^ In 2016, using similar data from the CAMARADES data set (mean group size=8), it was calculated that the statistical power of the preclinical stroke literature is only 45%, implying that half of the studies investigating a hypothesis which was correct would fail to find statistical evidence to support this. This is still, however, much larger than the 23% median power calculated across >700 neuroscience experiments.^59^ This problem is not unique to preclinical research. One review of data held within the Cochrane Reviews data set concluded that in 70% of 14 886 meta-analyses, all the publications included in the analyses were underpowered.^60^ Because small underpowered studies have a low chance of detecting a real effect, the statistical power of an experiment is a critical determinant of its value to users of the data. Meta-analysis can overcome this individual lack of power.

However, small sample size is also associated with more insidious risks. Publication bias is not the only possible explanation for funnel plot asymmetry. Small-study effects, where smaller studies show greater effects than larger studies are also detected this way. In both clinical and preclinical settings, this can occur because of heterogeneity between subjects in different sized studies.^50 61^ Such heterogeneity can occur for many reasons. For example, a small hospital-based study may not provide as good a representation of patients receiving a treatment as a large population-based study. Moreover, the hospital patients may have additional undeclared reasons for their selection into the study. In preclinical studies, it has been argued that heterogeneity between studies can be a good thing if it broadens the biological base of a series of experiments to improve generalisability.^62^ The caveat is that this benefit is only realised when research teams cooperate in data pooling or multicenter studies or when meta-research is conducted. If the heterogeneity is present, for example, because of attrition bias due to selective reporting of results in a subset of studies, it is clearly not beneficial. One study shows that attrition bias can inflate effect sizes by 25%–175% with the inflation greatest for the smallest studies.^63^ Moreover, because biases tend to co-occur in small studies, they have a disproportionate chance of being published and providing a falsely positive impression in preclinical meta-analyses.^64^ These issues leave the systematic reviewer with a number of important quandaries; when is the body of evidence big enough for systematic review or meta-analysis to add value to a field of research; do imperfect studies provide value despite their imperfections; should small studies be excluded because of an assumption of bias or is such an assumption an even bigger risk to finding the truth? At present these questions remain unanswered.

### Lesson 8: promising reviews are rarely followed-up by their authors

Within the 20 preclinical therapeutic meta-analyses we randomly selected, examination of the citations of these papers within PubMed (11 May 2021) revealed that many had been extensively cited. For example; 91 citations^29^; 44 citations^14^; 27 citations^25^; 24 citations^30^. However, for most, the citations provided no evidence of direct experimental follow-up (preclinical or clinical) of the principal findings by the systematic review team (15/20). While lack of time for follow-up is one potential explanation for this, especially with the more recent reviews, a disturbing alternative is that many systematic reviewers are not embedded within the experimental science teams where the knowledge they generate would have most value. These reviews may have been undertaken simply for the sake of doing a study, instead of for their most valuable purpose, to inform biological knowledge and improve experimental studies.

Moreover, while most authors (65%) concluded that the intervention being studied was effective, few (20%) also concluded that the evidence available was robust (table 3). This demonstrates that systematic reviewers are aware that the quality of their data does not lend itself to robust conclusions. We use the term robust here in the sense defined in the Oxford English Dictionary—strong; able to survive being used a lot and not likely to break—in order to capture the gestalt of a range of quality/risk of bias scoring systems and author conclusions that point towards an interpretation that the authors believed their analysis to have sufficiently answered their question or not. This robustness was our conclusion from their reviews and is of necessity subjective. However, it is perturbing that these studies do not provoke further research, as this might lend more weight to these positive findings and possibly lead to effective therapeutics being carried through to clinical trials. Where a team conducting a systematic review is able to interact with teams conducting the primary research, the quality and impact of subsequent research is enhanced (see for example, McCann *et al*^65^).

**Table 3:** Conclusions drawn by authors of systematic reviews on the effectiveness of studied therapies and the robustness of the included data, n=20

	Total reviews	% of reviews	References
Conclusion drawn on treatment			
Effective	13	65	12 13 16 17 19 22–24 26 27 29–31
Unsure	6	30	14 15 18 21 25 28
Not effective	1	5	20
Conclusion drawn on robustness of work			
Robust evidence, results could be refined further but is not essential	4	20	17 19 29 31
More evidence would be beneficial	8	40	12 13 20 22–24 27 30
Not enough evidence, more needed	7	35	14–16 21 25 26 28
Not reported	1	5	18

## Conclusions

Preclinical systematic review and meta-analysis are in its infancy and, like preclinical science itself, are busy absorbing lessons already learnt by other fields and earlier generations of scientists. The lessons presented above highlight considerations of the conduct of systematic reviews and where they can be improved to provide more informative information for the many scientific fields these reviews are important for. Data overload is common to all modern science and is a symptom of its successes. Dealing with that data overload requires a number of strategies. In the short term, we can ask ever more specific questions, but this focus occurs at the expense of the generalisability of our conclusions. We must build better ways of storing, indexing, retrieving and ensuring the availability of the data we generate. For meta-research in particular, it is important to be able to readily find and extract the data from its accompanying commentary. While systematic review and meta-analysis have played an important part in identifying the presence and importance of a variety of biases in the preclinical literature, until they are eliminated by better experimental conduct and reporting, we need to remain vigilant to their potential influence within our reviews. The recent advance in pre-registration of preclinical studies may help with improved experimental conduct and reporting. Moreover, because the foundations of our field include exposing the influence of poor scientific behaviours, we should adopt only the highest standards for our own work and expect that, as we learn more, these standards are likely to become more rigorous across the broader field of science. These increased standards would provide greater definiteness to the conclusions reached by systematic review and meta-analysis.

## Data Availability

Data are available in a public, open access repository.
